# Pathological Pathways of Olfactory Neuroblastoma: From Molecular Mechanisms to Targeted Therapy: A Narrative Review

**DOI:** 10.3390/cancers18152510

**Published:** 2026-08-05

**Authors:** Wenqiao Zhou, Xingchen Liu, Junying Hu, Yu Chen, Feng Liu, Bing Zhong

**Affiliations:** Department of Otolaryngology Head and Neck Surgery, West China Hospital of Sichuan University, 37Guoxue Lane, Chengdu 610041, China; wenqiaozhouyaoyao@163.com (W.Z.); 2246097527@qq.com (X.L.); hujunying2001@163.com (J.H.); 18141733402@163.com (Y.C.)

**Keywords:** olfactory neuroblastoma, molecular heterogeneity, molecular subtype, targeted therapy, tumor microenvironment, precision oncology

## Abstract

Olfactory neuroblastoma (ONB), also known as esthesioneuroblastoma, is a rare malignant tumor arising from the olfactory epithelium of the sinonasal tract. Surgery combined with radiotherapy remains the standard treatment for localized disease, whereas chemotherapy is mainly used in advanced or recurrent cases. However, recurrent and metastatic ONB continues to present major therapeutic challenges, and traditional staging and histological grading systems cannot fully explain the marked differences in clinical behavior among patients. The primary objective of this review is to summarize recent advances in the molecular pathology, tumor microenvironment (TME), and emerging targeted therapeutic strategies in ONB. Emerging genomic and transcriptomic studies suggest that ONB comprises biologically heterogeneous tumors with distinct molecular and transcriptional programs associated with proliferation, neuroendocrine differentiation, angiogenesis, and stromal remodeling. Furthermore, we explore the increasing attention directed toward the TME, including immune-cell infiltration, angiogenic signaling, and immune checkpoint expression, which may influence therapeutic response. These molecular findings have generated interest in several potential targeted treatment strategies, including peptide receptor radionuclide therapy (PRRT), anti-angiogenic therapy, epigenetic-targeted therapy, immunotherapy, and DNA-damage-response-targeted approaches. Ultimately, although the current evidence remains limited because of the rarity of the disease, novel therapeutic strategies for ONB are emerging. In addition to summarizing the current landscape, this review discusses the translational challenges and future directions for precision oncology and biomarker-driven therapy, aiming to provide insights for improving individualized patient management.

## Simple Summary

Olfactory neuroblastoma (ONB) is a rare cancer that develops in the upper part of the nasal cavity and can behave very differently among patients. Although surgery and radiotherapy remain the main treatments, some patients experience tumor recurrence or spread, for which effective treatment options are limited. Recent research has revealed that ONB is not a single uniform disease but consists of tumors with different biological characteristics. This review summarizes how advances in molecular research have improved our understanding of ONB and discusses how these discoveries may contribute to the development of more personalized treatments. We also highlight current challenges, including the need for better biomarkers and larger clinical studies, to improve future management of patients with this rare tumor.

## 1. Introduction

Olfactory neuroblastoma (ONB), also referred to as esthesioneuroblastoma, is a rare malignant neuroectodermal neoplasm arising from the olfactory neuroepithelium within the superior nasal cavity. ONB accounts for approximately 2–6% of all sinonasal malignancies. While it can occur at any age, the incidence shows a peak in patients between 40 and 60 years old, with equal prevalence between males and females. And ONB demonstrates a broad spectrum of clinical behavior ranging from indolent localized disease to highly aggressive tumors with local invasion, recurrence, and distant metastasis [1,2,3]. Because of its rarity, the biological characteristics and optimal therapeutic management of ONB remain incompletely understood.

Current standard treatment for ONB generally involves multimodal therapy, consisting of surgery combined with radiotherapy, with chemotherapy reserved for advanced, recurrent, or metastatic disease [4,5,6]. Craniofacial resection and endoscopic skull-base surgery have improved local disease control, while intensity-modulated radiotherapy and stereotactic techniques have enhanced treatment precision. Nevertheless, recurrence rates remain substantial, particularly in advanced-stage tumors, and long-term management of recurrent or treatment-refractory ONB continues to represent a major clinical challenge [7,8].

Current prognostic assessment of olfactory neuroblastoma mainly relies on clinical staging systems, including the modified Kadish and Dulguerov classifications, together with Hyams histological grading. Recent multicenter studies and meta-analyses have confirmed their prognostic significance for survival and recurrence outcomes [9,10,11]. The Kadish system stratifies tumors based on anatomical extension, while the Hyams grading system evaluates histopathological differentiation, mitotic activity, necrosis, and neurofibrillary matrix formation. Although these classification systems remain clinically valuable, they do not fully explain the marked heterogeneity observed in treatment response and clinical outcome. Patients with similar pathological grades may have substantially different disease trajectories, which shows the presence of underlying molecular and biological diversity.

Over the past decade, advances in genomic sequencing and molecular profiling have improved the understanding of the biological characteristics of ONB [12,13]. Recent genomic and transcriptomic studies have revealed substantial molecular heterogeneity and identified several potentially actionable alterations [14,15,16]. Although the clinical implications of these findings are still being clarified, they provide rational evidence to explore molecularly guided therapeutic approaches in ONB.

The TME is increasingly recognized as an important component of ONB biology. Recent studies have identified heterogeneous patterns of immune-cell infiltration, stromal immune localization, and immune checkpoint expression across ONB specimens [17,18,19]. Emerging evidence also suggests that immunosuppressive microenvironmental features may contribute to disease progression and therapeutic resistance [20,21]. Accordingly, immunotherapeutic approaches and TME-directed strategies are being explored in recurrent or advanced ONB.

Patients with recurrent or metastatic ONB often have limited systemic treatment options, and there is no standardized targeted therapeutic strategy. Despite these advances, evidence supporting targeted therapies in ONB remains limited because of the rarity of the disease and the lack of large prospective studies. Current knowledge is derived largely from retrospective analyses, small case series, and extrapolation from other neuroendocrine malignancies. Nevertheless, continued progress in molecular profiling and translational research has improved the understanding of the biological heterogeneity of ONB and may facilitate the development of biomarker-guided therapeutic strategies [22].

To better understand this complexity of ONB, this review first presents a biological classification framework for ONB. This review summarizes recent advances in the molecular biology of ONB, with emphasis on molecular heterogeneity, emerging therapeutic targets, and future translational opportunities. To provide a comprehensive overview of the molecular landscape and multimodal management of ONB, we conducted a literature search of PubMed/MEDLINE and Web of Science databases for articles published up to 2026. The search strategy utilized keywords including esthesioneuroblastoma, olfactory neuroblastoma, genomics, molecular pathogenesis, and targeted therapy. We prioritized recent, high-impact original research and relevant reviews to highlight emerging trends while also referencing foundational studies for epidemiological context. Studies were selected based on their relevance to the scope of this review, with priority given to publications providing molecular, translational, or clinical evidence directly related to ONB. No formal systematic review methodology was applied because this article is a narrative review. Given the rarity of ONB, pivotal studies on extrapulmonary neuroendocrine carcinomas and small-cell lung cancer (SCLC) were also included to elucidate potential shared molecular mechanisms and therapeutic implications.

## 2. Molecular Subclassification and Biological States in ONB

**Figure 1:**
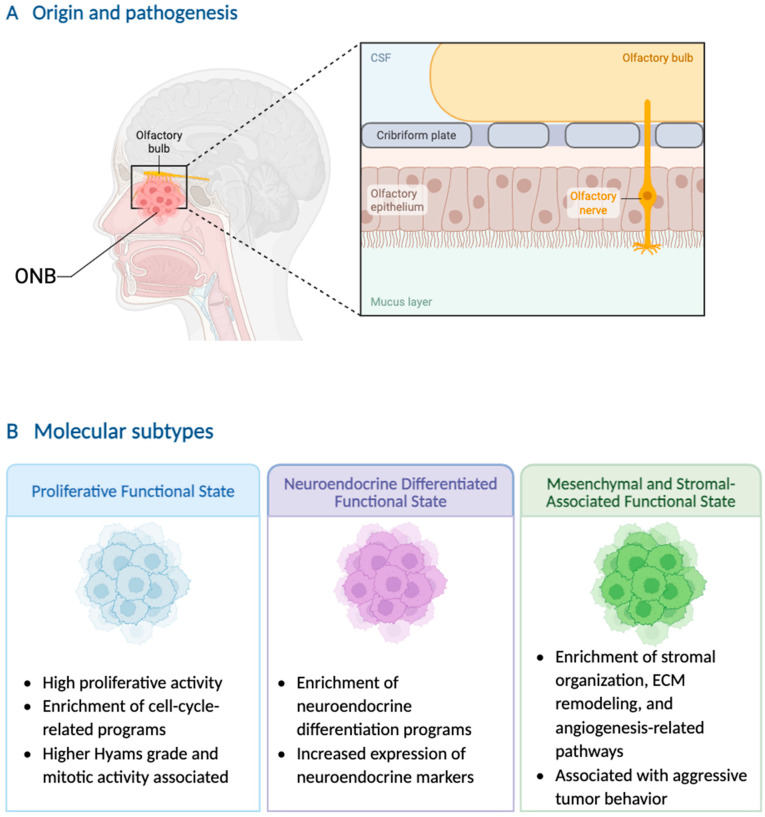
Origin, pathogenesis, and proposed molecular functional states of olfactory neuroblastoma (ONB). Created with BioRender.com. (**A**) Schematic illustration of the proposed origin and pathogenesis of ONB arising from the olfactory epithelium adjacent to the cribriform plate and olfactory bulb. (**B**) Emerging molecular functional states identified in ONB: the Proliferative state (high cell-cycle activity), the Neuroendocrine-Differentiated state (enriched neuroendocrine programs), and the Mesenchymal/Stromal-Associated state (linked to stromal remodeling and aggressive behavior). These molecular functional states are proposed and have not yet been clinically validated.

Traditional classification systems for ONB are primarily based on anatomical extent and histopathological features but do not fully capture the biological heterogeneity of the disease. Recent genomic and transcriptomic studies have identified distinct transcriptional programs associated with proliferative activity, neuroendocrine differentiation, and stromal or mesenchymal signaling, suggesting the existence of biologically diverse functional states within ONB [14,15,16]. In this review, we integrate these observations into a proposed conceptual framework comprising three molecular functional states—Proliferative, Neuroendocrine-Differentiated, and Mesenchymal/Stromal-Associated—which is intended to facilitate interpretation of the current molecular evidence rather than to represent a validated molecular classification system (Figure 1). Furthermore, the stromal- and angiogenesis-associated transcriptional signatures identified in current transcriptomic studies may reflect stromal or microenvironment-associated signals rather than conclusively demonstrating a tumor-cell-intrinsic mesenchymal differentiation program. Therefore, the proposed Mesenchymal/Stromal-Associated state should be interpreted as a conceptual stromal-associated functional state whose biological basis requires further validation (Table 1).

### 2.1. Proliferative Functional State

Transcriptomic studies have identified ONB subgroups exhibiting proliferative transcriptional programs and progenitor-like characteristics. In this review, these observations are conceptually integrated into a proliferative functional state rather than representing a formally established molecular subtype [14]. These tumors demonstrate elevated expression of genes associated with mitotic progression, DNA replication, and cellular proliferation [16].

Reference to the research results of SCLC, proliferative transcriptional states may correlate with more aggressive clinicopathologic features [16,23]. In some studies, tumors with increased proliferative signatures have been associated with higher Hyams grade, increased mitotic activity, necrosis, and reduced neurofibrillary matrix formation [23].

### 2.2. Neuroendocrine-Differentiated Functional State

Neuroendocrine differentiation is a defining feature of ONB and remains central to its pathologic diagnosis. However, recent transcriptomic studies suggest that the degree of neuroendocrine-associated signaling may vary across tumors [14,15].

Neuroendocrine-enriched ONB states demonstrate increased expression of markers and transcriptional regulators associated with neuroendocrine lineage differentiation, including synaptophysin, chromogranin A, INSM1, SSTR2, ASCL1, and NEUROD1. These tumors may share certain biological features with other neuroendocrine neoplasms [16,24,25].

Somatostatin receptor 2 (SSTR2) is frequently expressed in neuroendocrine-enriched ONB and represents one of the characteristic markers of this transcriptional state. Beyond its diagnostic value, SSTR2 also provides the biological rationale for receptor-targeted imaging and peptide receptor radionuclide therapy, which are discussed in Section 5.1.

### 2.3. Mesenchymal and Stromal-Associated Functional State

Transcriptomic analyses have identified subsets of ONB demonstrating increased expression of genes associated with stromal organization, extracellular matrix (ECM) remodeling, and angiogenesis-related pathways [14,16]. These findings suggest that certain tumors may exhibit microenvironmental and stromal features distinct from more neuroendocrine-differentiated states.

The biological significance of mesenchymal or stromal-associated transcriptional programs in ONB remains incompletely understood. However, stromal enrichment and angiogenesis-related signaling have been associated with aggressive behavior in several solid malignancies and may contribute to intertumoral heterogeneity in ONB [26,27].

Angiogenesis-related pathways have attracted particular interest in this context. Some studies have reported increased microvessel density and angiogenesis-associated marker expression in ONB, especially in advanced disease [28,29]. Nevertheless, clinical evidence supporting anti-angiogenic therapy in ONB remains limited.

TME composition may also influence immune-cell infiltration and therapeutic response. Although stromal remodeling and immune exclusion have been described in other malignancies, comparable mechanisms in ONB have not yet been well characterized [30,31,32].

However, these transcriptional states do not exist in isolation; they are likely driven by specific genomic alterations and maintained by a supportive TME. To fully understand the biological basis of these subtypes, we must look beyond the transcriptome to identify the upstream drivers.

Although these transcriptional states provide a conceptual framework for understanding ONB heterogeneity, current evidence remains insufficient to establish them as validated molecular subtypes. They should therefore be regarded as emerging transcriptional programs reflecting distinct functional states rather than definitive clinical classifications. Future studies integrating transcriptomic, genomic, and clinical data will be required to determine their biological and clinical relevance [16,33,34].

## 3. Molecular Landscape of ONB

**Figure 2:**
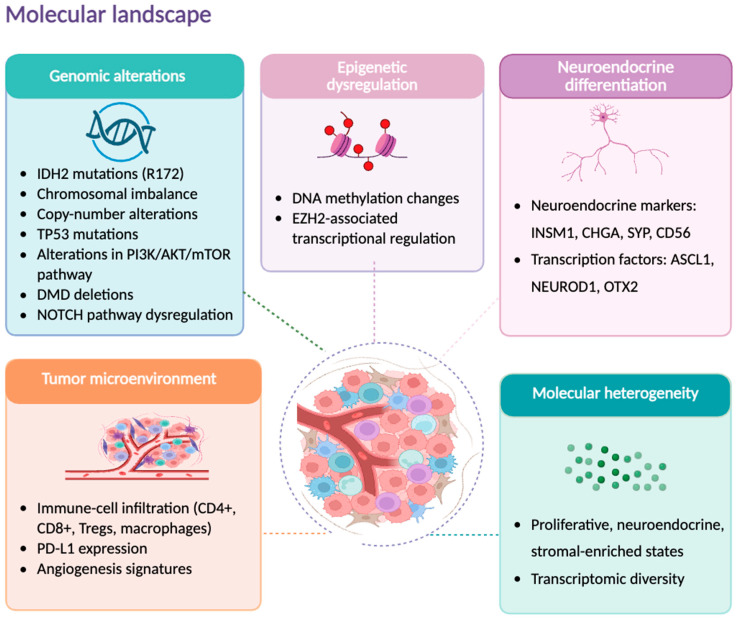
Overview of the molecular landscape in olfactory neuroblastoma (ONB). Created with BioRender.com. Summary of the molecular landscape of ONB, including genomic alterations, epigenetic dysregulation, neuroendocrine differentiation, tumor microenvironmental features, and molecular heterogeneity.

Building upon the biological framework established in the previous section, we now explore the upstream genomic and epigenomic mechanisms that orchestrate these distinct molecular subtypes (Figure 2).

### 3.1. Genomic Alterations in ONB

Compared with more common solid tumors, the genomic landscape of ONB remains poorly characterized, largely because of its rarity and the limited availability of large sequencing cohorts. Nevertheless, recent molecular studies have identified several recurrent alterations that may play a role in ONB pathogenesis [35].

Recurrent IDH2 mutations, particularly involving the R172 hotspot, have been reported in a subset of sinonasal tumors historically diagnosed as ONB and represent one of the most notable molecular alterations associated with this diagnostic spectrum [12,36]. Mutant IDH2 promotes accumulation of the oncometabolite 2-hydroxyglutarate (2-HG), which has been linked to epigenetic dysregulation, altered DNA methylation, and impaired cellular differentiation [37,38]. The identification of IDH2 alterations has generated interest in the potential application of mutant-IDH inhibitors; however, such therapeutic implications remain exploratory in sinonasal tumors and should be interpreted cautiously until validated in molecularly confirmed ONB cohorts [22]. However, the interpretation of IDH2 R172 mutations in ONB requires caution. Recent molecular studies have suggested that some IDH2-mutant sinonasal tumors, including cases historically diagnosed as SNUC or poorly differentiated sinonasal carcinoma, may represent a distinct molecular entity rather than conventional ONB [12,39]. Therefore, some previously reported IDH2-mutant ONB cases may warrant reassessment based on integrated morphological, immunophenotypic, and molecular features, and whether IDH2 R172 mutations represent a bona fide ONB driver remains unresolved. Accordingly, IDH2 R172 mutations should not currently be regarded as a defining genomic alteration in conventional ONB, and molecular findings from IDH2-mutant sinonasal tumors should be interpreted with caution until validated in diagnostically confirmed ONB cohorts.

Other reported genomic abnormalities include TP53 mutations, alterations affecting the PI3K/AKT/mTOR signaling pathway, DMD deletions, and NOTCH pathway dysregulation [13,19,40]. However, no single dominant oncogenic driver has been consistently identified across published cohorts, suggesting substantial molecular heterogeneity among ONB tumors.

Copy-number alterations appear to be more frequent in biologically aggressive tumors. Several studies using comparative genomic hybridization have identified recurrent chromosomal gains and losses in ONB, which suggests underlying chromosomal instability [41,42]. Some reports have suggested associations between chromosomal instability and adverse clinical behavior, although these findings remain limited by small sample size and cohort heterogeneity [43,44].

In contrast to sinonasal undifferentiated carcinoma and several other head and neck malignancies, ONB generally exhibits a relatively low tumor mutational burden (TMB) [16,45].

Taken together, the available data support the concept that ONB comprises a biologically heterogeneous group of tumors rather than a single molecular entity [16] (Table 2).

### 3.2. Epigenetic Dysregulation

Epigenetic dysregulation has been increasingly implicated in the molecular biology of ONB. Alterations involving DNA methylation, chromatin remodeling, and transcriptional regulation have been reported in recent molecular profiling studies and may contribute to neuroendocrine differentiation and tumor heterogeneity [35,36,48].

Methylation profiling analyses suggest that ONB has epigenetic features distinct from those of other sinonasal malignancies [52]. These findings support the possibility that epigenetic alterations contribute to tumor classification and biological behavior. In addition, tumors harboring IDH2 mutations may demonstrate altered methylation patterns associated with impaired cellular differentiation and transcriptional dysregulation [53].

Epigenetic regulators such as enhancer of zeste homolog 2 (EZH2) have also been implicated in several neuroendocrine malignancies. EZH2-associated chromatin remodeling has been linked to tumor progression and lineage plasticity in other neuroendocrine tumor types, such as in treatment-induced neuroendocrine prostate cancer [36,46]. However, there is still lack of distinct evidence supporting a comparable role in ONB.

Emerging evidence from other malignancies further suggests that epigenetic alterations may influence immune signaling and antigen presentation [54,55]. Although these mechanisms have not been well characterized in ONB, they have generated interest in the potential integration of epigenetic therapies with immunotherapeutic approaches in recurrent or advanced disease [56,57].

### 3.3. Neuroendocrine-Associated Transcription Factors

ONB characteristically expresses multiple neuroendocrine lineage markers and transcriptional regulators. Conventional neuroendocrine markers, including synaptophysin, chromogranin A, CD56, and INSM1, are frequently expressed and remain important in routine pathologic diagnosis [50].

Recent transcriptomic studies have further identified differential expression of developmental transcription factors associated with neural and neuroendocrine differentiation, including OTX2, ASCL1, and NEUROD1 [14,15]. Among these, OTX2 has attracted particular interest because of its established role in neuroectodermal development and neuronal lineage specification. Increased OTX2 expression has been reported in ONB and may contribute to maintenance of neuroendocrine differentiation programs [22].

ASCL1 and NEUROD1 are well-recognized regulators of neuroendocrine lineage states in SCLC and other neuroendocrine malignancies [33,49]. Their expression in ONB further supports the neuroendocrine phenotype of the tumor, although their precise biological and therapeutic significance in ONB remains unclear.

INSM1 has recently emerged as a sensitive marker of neuroendocrine differentiation in ONB and other neuroendocrine neoplasms [50,58]. In addition to its diagnostic utility, INSM1 expression may reflect underlying lineage-specific transcriptional programs involved in neuroendocrine differentiation [59]. Collectively, neuroendocrine markers (synaptophysin, chromogranin A, CD56, INSM1) and lineage-associated transcription factors (OTX2, ASCL1, NEUROD1) define a coordinated neuroendocrine regulatory network in ONB (Table 3), reflecting both diagnostic and biological dimensions of neuroendocrine differentiation.

Beyond their diagnostic utility, these molecular alterations—ranging from transcriptional dysregulation to specific genomic mutations—carry significant biological implications. For instance, the epigenetic reprogramming driven by IDH2 mutations and the genomic instability associated with TP53 pathway abnormalities may directly influence tumor progression and therapeutic response [41,46]. Consequently, these features represent promising targets for precision medicine approaches.

Similarly, recurrent copy-number alterations contribute to the molecular heterogeneity of ONB. While the biological significance of individual alterations requires further elucidation, increasing genomic instability is generally associated with aggressive behavior, suggesting that the burden of genomic alterations may serve as a prognostic indicator [37,41,43].

Collectively, these genomic, epigenetic, and transcriptional alterations highlight the molecular complexity of ONB and provide a foundation for understanding its biological heterogeneity. Several of these molecular features have also emerged as potential therapeutic targets and will be discussed in later sections.

## 4. Tumor Microenvironment and Immune Landscape in ONB

### 4.1. Immune-Cell Infiltration Patterns

Increasing evidence suggests that the TME contributes to the biological heterogeneity of ONB by influencing immune composition, stromal interactions, and local signaling networks. Available studies have demonstrated variable infiltration of T lymphocytes, macrophages, dendritic cells, and other immune cell populations across tumors [34,60,61].

Several immunohistochemical studies have identified CD8-positive T-cell infiltration in subsets of ONB, indicating the presence of adaptive immune responses within the TME [17,19]. However, the extent and composition of immune infiltration appear to vary considerably among individual tumors [62].

Recent genomic and immune profiling studies have identified varying proportions of dendritic cells, macrophages, and other innate immune populations, suggesting distinct immune microenvironmental states characterized by different levels of immune activation or suppression. In addition to cytotoxic T lymphocytes, varying proportions of dendritic cells, macrophages, and other innate immune populations have been identified. These differences may contribute to distinct immune microenvironmental states characterized by varying levels of immune activation or suppression [17,19].

Furthermore, recent investigations have suggested that expression of immune-related molecules including HLA-DR, CXCL9, and CXCL10 may influence antitumor immune responses in ONB. These observations raise the possibility that immunological biomarkers could eventually assist in identifying patients most likely to benefit from immunotherapeutic approaches [17,19].

Tumor-associated macrophages (TAMs) and other stromal immune populations may also participate in microenvironmental regulation in ONB. Although macrophage-associated immunosuppressive signaling has been described in other solid malignancies, its biological significance in ONB has not completely been clarified [31,63].

### 4.2. PD-L1 Expression and Immune Checkpoint Signaling

PD-L1 expression has been evaluated as a potential indicator of immune checkpoint pathway activation in ONB. Reported PD-L1 positivity rates vary substantially among studies, likely because of differences in detection methods, scoring systems, positivity thresholds and cohort composition [64]. In addition, biological heterogeneity, including differences in tumor grade, disease stage, and intratumoral immune composition, may further contribute to the observed variability. Therefore, direct comparison of PD-L1 positivity rates across studies should be interpreted with caution. A subset of ONB tumors demonstrates PD-L1 expression accompanied by immune-cell infiltration, showing the presence of an immunologically active TME in some cases. However, the clinical significance and predictive value of PD-L1 expression in ONB are still undetermined because of limited data [51,65,66]. This variability is likely multifactorial and may reflect differences in PD-L1 expression, tumor immune microenvironment, patient selection, and the small sample sizes of the currently available studies. ONB generally appears to exhibit a relatively low TMB compared with several other immunotherapy-responsive malignancies, consistent with findings from recent genomic and transcriptomic studies [16,35,42]. However, in addition to TMB, factors including TME composition, immune-cell infiltration, and immune-related signaling pathways may also contribute to variability in immunotherapy response, highlighting the need for more comprehensive biomarker evaluation beyond PD-L1 alone. Although the composition of the immune microenvironment has been described in several ONB studies, the molecular mechanisms governing immune-cell recruitment, macrophage polarization, and immune suppression remain poorly understood. Whether signaling pathways involving chemokines, cytokines, or macrophage-polarizing factors contribute to immune escape in ONB has not yet been systematically investigated. Clinical implications are discussed in Section 5.4.

### 4.3. Angiogenesis and Stromal Remodeling

Angiogenesis appears to contribute to the biological behavior of ONB, particularly in advanced or high-grade tumors. Increased vascularity and elevated expression of angiogenesis-associated markers, including VEGF, have been reported in subsets of aggressive disease [28]. Stromal composition and ECM organization may also influence tumor architecture and microenvironmental heterogeneity in ONB. However, the mechanisms underlying stromal remodeling in ONB remain poorly characterized. Emerging transcriptomic studies suggest that some ONB tumors demonstrate relative enrichment of stromal and angiogenesis associated signaling pathways [14,16]. Nevertheless, the biological and therapeutic significance of these observations is not clear.

In addition to VEGF-mediated angiogenic signaling, recent studies have suggested that angiopoietin-like 4 (ANGPTL4) may also be involved in tumor progression and adverse outcomes in several malignancies [67]. Although direct evidence in olfactory neuroblastoma remains limited, ANGPTL4 has been implicated in promoting angiogenesis, vascular permeability, and metastatic potential in other tumor types. Whether ANGPTL4 overexpression contributes to aggressive behavior or poor prognosis in ONB remains to be clarified and warrants further investigation [68].

### 4.4. Spatial Organization of the TME

Recent advances in spatial transcriptomics have highlighted the importance of spatially organized cellular interactions in shaping tumor progression and therapeutic response. Unlike conventional bulk sequencing approaches, spatial transcriptomic technologies preserve tissue architecture and enable the simultaneous characterization of gene expression and cellular localization within the tumor ecosystem. Although dedicated spatial transcriptomic studies in ONB remain limited, emerging evidence suggests that substantial intratumoral heterogeneity exists across both malignant and non-malignant cell populations. Such heterogeneity may influence immune surveillance, tumor invasion, and treatment sensitivity [16,22].

TAMs represent a key component of the ONB microenvironment. Depending on their polarization state, TAMs may exert either anti-tumor or pro-tumor functions through regulation of angiogenesis, ECM remodeling, immune suppression, and cytokine secretion. Studies in other solid tumors have demonstrated that macrophage-rich microenvironments are frequently associated with disease progression and resistance to immunotherapy; whether similar mechanisms operate in ONB remains unclear. Characterizing the spatial distribution and functional states of TAMs in ONB may therefore provide novel therapeutic opportunities [30,63].

In addition to immune cells, stromal components including cancer-associated fibroblasts, ECM proteins, and growth factor signaling pathways contribute substantially to tumor behavior. Stromal signaling networks involving bone morphogenetic protein (BMP)-related molecules, transforming growth factor-β (TGF-β), and ECM remodeling have been implicated in tumor progression across several malignancies. Whether similar stromal programs contribute to ONB aggressiveness, metastatic dissemination, or therapeutic resistance remains largely unknown using integrated spatial and single-cell approaches [21,26,27].

## 5. Current and Emerging Targeted Therapeutic Strategies

The management of olfactory neuroblastoma is primarily based on surgical resection combined with radiotherapy, which remains the standard of care across most stages of disease. Systemic chemotherapy is reserved for advanced or metastatic cases, although optimal regimens are not well established. In contrast, most targeted and immune-based therapeutic approaches discussed in this section remain investigational and are supported mainly by small retrospective studies, case reports, or extrapolation from other neuroendocrine malignancies. Therefore, the following subsections summarize emerging strategies in ONB within a framework of current evidence levels and translational relevance.

### 5.1. Peptide Receptor Radionuclide Therapy

PRRT has attracted increasing interest as a treatment option for recurrent or metastatic ONB because many tumors demonstrate somatostatin receptor expression, particularly SSTR2. Somatostatin receptor imaging using gallium-68 DOTATATE or DOTANOC PET/CT has demonstrated utility for disease detection, staging, and treatment evaluation in selected ONB patients [69,70,71]. Tumors with high somatostatin receptor uptake may be considered for radionuclide-based therapy. Lutetium-177 DOTATATE is an established treatment for several neuroendocrine tumor types and has also been explored in ONB [71,72,73,74,75]. Published experience in ONB is largely limited to case reports and small retrospective series, although radiographic responses and disease stabilization have been anecdotally reported in selected patients, the objective response rate and long-term efficacy remain poorly defined due to the heterogeneity in dosing and patient selection.

The biological rationale for PRRT in ONB is supported by the frequent expression of SSTR2, which is observed in a substantial proportion of tumors. In addition to providing a therapeutic target, SSTR2 expression may also serve as a biomarker for patient selection [45,48]. Functional imaging using radiolabeled somatostatin analogues allows for assessment of receptor expression throughout the disease burden and may facilitate individualized treatment planning [54]. In addition, 18F-FDG PET-CT may provide complementary information for tumor metabolic assessment and radiotherapy planning in selected patients, particularly in higher-grade or more aggressive disease [76,77].

**Figure 3:**
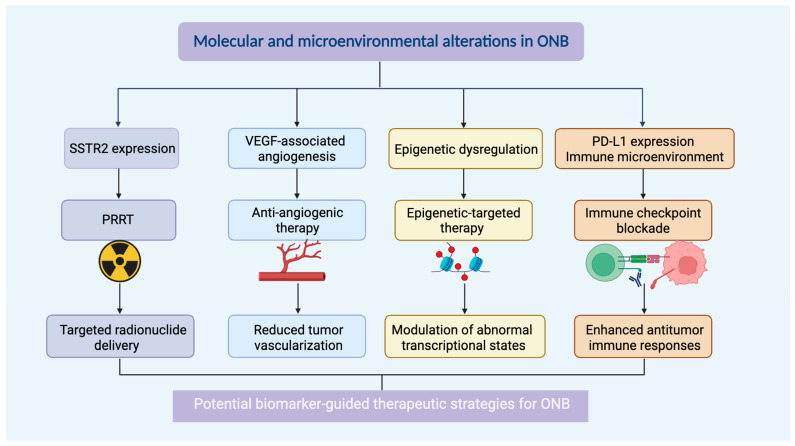
Mechanistic framework of emerging targeted therapeutic strategies in olfactory neuro blastoma (ONB). Molecular alterations and tumor microenvironmental features identified in ONB provide the biological rationale for several emerging therapeutic approaches. From left to right, the figure illustrates: (1) SSTR2 expression supports peptide receptor radionuclide therapy (PRRT) through receptor-directed radionuclide delivery; (2) VEGF-associated signaling provides the basis for anti-angiogenic strategies targeting tumor vascularization; (3) epigenetic dysregulation providing the rationale for epigenetic-targeted approaches aimed at modulating abnormal transcriptional states; and (4) PD-L1 expression and immune microenvironmental features supporting immune checkpoint blockade to enhance antitumor immune responses. Together, these biomarker-guided strategies represent potential precision therapeutic approaches for ONB. Representative therapeutic agents and the current clinical evidence are summarized in Table 4.

Although prospective studies are lacking, available reports suggest that PRRT may be considered as a therapeutic option for carefully selected patients with recurrent or metastatic SSTR-positive ONB, where durable disease stabilization has been reported in some cases. The favorable toxicity profile observed in other neuroendocrine tumors further supports continued evaluation of this strategy in ONB [52,53] (Figure 3 and Table 4).

### 5.2. Anti-Angiogenic Therapy

Angiogenesis-related signaling has been implicated in subsets of ONB, particularly in advanced or high-grade tumors demonstrating increased vascularity and VEGF expression [28]. These observations have generated interest in anti-angiogenic therapeutic approaches. Clinical experience with anti-angiogenic therapy in ONB is scarce. Bevacizumab-based therapy has been reported in isolated cases of recurrent or metastatic ONB [29]. Although anti-angiogenic therapy has been explored in combination with chemotherapy or immunotherapy in other malignancies, its role in ONB has not yet been clearly established.

### 5.3. Epigenetic-Targeted Therapy

Epigenetic dysregulation contributes to the molecular heterogeneity of sinonasal neuroendocrine malignancies, with IDH2 mutations representing a potentially relevant but diagnostically complex example. In tumors harboring confirmed IDH2 alterations, mutant IDH2-mediated accumulation of 2-HG provides a biological rationale for considering epigenetic-targeted strategies. However, direct preclinical evidence supporting epigenetic-targeted therapy in ONB remains scarce, and most currently available data are extrapolated from studies of other neuroendocrine and IDH-mutant malignancies. Therapeutic strategies directed at chromatin remodeling, histone modification, and DNA methylation pathways have demonstrated activity in several neuroendocrine and solid malignancies; however, their relevance to ONB remains largely extrapolative and requires disease-specific validation [78,79]. Preclinical studies in other tumor types have suggested that epigenetic-modifying agents may influence tumor differentiation states and modulate immune-related signaling pathways, thereby potentially enhancing tumor immunogenicity and antitumor immune responses [54].

Altered DNA methylation patterns, histone modifications, and dysregulated activity of epigenetic regulators such as EZH2 may contribute to maintenance of malignant transcriptional programs. Although direct clinical evidence in ONB remains limited, there are currently no published preclinical studies demonstrating the efficacy of EZH2 inhibitors or DNA methylation-targeting agents in established ONB models. Likewise, no validated epigenetic biomarkers have yet been identified to predict therapeutic response in ONB [80].

Mutant IDH2 catalyzes the abnormal conversion of α-ketoglutarate (α-KG) to the oncometabolite 2-HG, resulting in intracellular accumulation of 2-HG. Elevated 2-HG competitively inhibits α-KG-dependent dioxygenases, including TET DNA demethylases and Jumonji-domain histone demethylases, leading to widespread DNA and histone hypermethylation. This hypermethylator phenotype, analogous to the glioma CpG island methylator phenotype (G-CIMP) described in IDH-mutant tumors, is thought to contribute to aberrant transcriptional regulation, impaired cellular differentiation, and tumorigenesis. Whether an analogous methylation phenotype exists in ONB remains unknown. Although a comparable methylation phenotype has not been fully characterized in bona fide ONB, IDH2-mutant sinonasal tumors demonstrate epigenetic consequences consistent with altered 2-HG metabolism. Whether these mechanisms contribute to tumor biology in diagnostically confirmed ONB requires further investigation [37,38,81].

At present, there is insufficient evidence to determine whether IDH2-mutant ONBs preferentially correspond to any of the emerging transcriptional states described in recent transcriptomic studies. Future integrated genomic and transcriptomic analyses will be required to clarify this relationship.

Specific inhibitors targeting mutant IDH2, such as enasidenib, have been approved for the treatment of relapsed or refractory acute myeloid leukemia harboring IDH2 mutations. These agents selectively inhibit the neomorphic activity of mutant IDH2, thereby reducing intracellular 2-HG levels and promoting cellular differentiation through reversal of epigenetic dysregulation in AML. Although clinical evidence supporting the use of mutant IDH2 inhibitors in ONB is currently lacking, the high frequency of IDH2 R172 mutations provides a compelling biological rationale for evaluating these agents in molecularly selected patients through future prospective clinical studies [37,38,81]. In addition, several practical challenges remain to be addressed before IDH2-targeted therapy can be translated into ONB, including the absence of disease-specific preclinical models, limited clinical experience, and the need to better define the pharmacologic and clinical feasibility of these agents in this rare malignancy.

Beyond epigenetic-targeted approaches, other actionable genomic alterations may also provide opportunities for precision therapy in ONB. Alterations involving the PI3K/AKT/mTOR pathway have been reported in subsets of tumors, providing a biological rationale for investigating pathway-directed therapies [82]. mTOR inhibitors, including everolimus and sirolimus, have demonstrated activity in other malignancies with pathway activation; however, their therapeutic relevance in ONB remains hypothetical and requires validation in molecularly selected cohorts. Among these actionable alterations, FGFR3 amplification has emerged as one of the most directly targetable molecular abnormalities [83]. Although not yet widely implemented in clinical practice, the presence of FGFR3 amplification provides a strong rationale for exploring FGFR inhibitors (e.g., erdafitinib, pemigatinib) in biomarker-selected patients. These agents, already approved for other malignancies harboring FGFR alterations, represent a promising avenue for precision therapy in ONB, particularly in cases with limited treatment options.

Consequently, combination approaches integrating epigenetic therapy with immunotherapy in SCLC have emerged as an area of growing interest. Whether such strategies may be beneficial for patients with recurrent or advanced ONB remains an important question for future translational investigation [84]. At present, both preclinical and clinical evidence supporting epigenetic-targeted therapy in ONB remain extremely limited.

### 5.4. Immune Checkpoint Blockade

Immune checkpoint inhibitors have been explored in recurrent or metastatic ONB, although currently available clinical evidence remains limited. Case reports and small retrospective studies have described responses to anti-PD-1 therapy, including pembrolizumab and nivolumab, in selected patients with advanced disease [51,66,85]. However, treatment outcomes have been variable, and durable responses have not been consistently observed. Based on the current evidence, immune checkpoint inhibitors cannot yet be regarded as a standard treatment for ONB but may be considered on an individualized basis in selected patients with recurrent or metastatic disease, particularly when conventional treatment options are limited. The heterogeneous immune microenvironment of ONB may contribute to differences in immunotherapy response among tumors. Potential biomarkers under research include PD-L1 expression and patterns of immune-cell infiltration [86,87].

### 5.5. Translational Insights from Other Neuroendocrine Malignancies

Although ONB is biologically distinct from other neuroendocrine malignancies, some molecular features overlap with tumors such as small-cell lung cancer and neuroendocrine prostate cancer. Transcription factors including ASCL1 and NEUROD1, as well as epigenetic regulatory pathways, have been implicated across multiple neuroendocrine tumor types [33,49]. Therapeutic approaches developed for other neuroendocrine neoplasms, including PRRT and immune-based strategies, have therefore attracted interest in ONB.

Overall, while these emerging strategies provide promising biological rationale, their clinical applicability in ONB remains unproven, and robust prospective clinical trials are needed to define their therapeutic value.

## 6. Future Directions and Translational Challenges

Because ONB is a rare and biologically heterogeneous malignancy, current evidence remains limited by retrospective study designs, relatively small patient cohorts, and substantial variability in treatment strategies, pathological classification systems, and molecular testing approaches. Future progress will likely depend on multicenter and international collaborative efforts that facilitate larger patient cohorts, standardized pathological and molecular characterization, and more robust evaluation of clinical outcomes. Advances in molecular profiling, neuroendocrine differentiation markers, immune microenvironment analysis, single-cell sequencing, and spatial transcriptomics may further improve our understanding of ONB heterogeneity and identify clinically relevant biomarkers, including somatostatin receptor expression, PD-L1 expression, and subtype-associated molecular alterations [88]. In parallel, the development of biologically representative preclinical models, such as organoid systems and patient-derived xenografts, will be essential for investigating tumor biology and therapeutic response. Given the rarity of ONB, conventional randomized clinical trials remain challenging; therefore, basket-trial designs, precision oncology approaches, and inclusion of ONB patients in broader neuroendocrine tumor clinical trial programs may provide practical opportunities to evaluate emerging targeted and immunotherapeutic strategies. Ultimately, integrating molecular, immunological, and clinical data may help refine current subtype classifications and support the development of more individualized treatment strategies for ONB.

A key challenge moving forward is the integration of multi-omic datasets to construct a comprehensive, hierarchical model of ONB biology. Rather than viewing genomic, transcriptomic, and microenvironmental data in isolation, future research should focus on how these layers interact. For instance, how do specific copy-number alterations influence the epigenetic landscape, and how does the resulting transcriptomic profile shape the immune microenvironment? Addressing these questions through integrative bioinformatic analyses will be vital for identifying master regulators of tumor behavior and for distinguishing driver events from passenger alterations.

ONB, like many cancers, is not a static entity but evolves over time. Intratumoral heterogeneity (ITH) poses a significant barrier to effective treatment. Different regions of the same tumor may harbor distinct molecular profiles—a phenomenon known as spatial heterogeneity [89,90]. Furthermore, temporal heterogeneity arises as the tumor evolves under the selective pressure of treatment. This clonal evolution can lead to the emergence of resistant subclones that were minor or undetectable at diagnosis [91]. For example, a tumor may shift from a neuroendocrine-differentiated state to a more mesenchymal or treatment-resistant state following chemotherapy or radiotherapy. Recognizing this dynamic nature is crucial; a single biopsy may not capture the full molecular complexity of the disease. Therefore, liquid biopsies and longitudinal sampling represent promising avenues for monitoring clonal evolution and adapting treatment strategies in real-time [92].

## 7. Conclusions

Olfactory neuroblastoma is a rare sinonasal neuroendocrine malignancy characterized by substantial clinical and molecular heterogeneity. At present, the mainstay of treatment remains surgical resection combined with radiotherapy, while chemotherapy is selectively used in advanced or unresectable disease. Metastatic spread typically involves regional cervical lymph nodes and distant organs such as the lung, bone, and liver, with more aggressive dissemination patterns observed in high-grade or advanced-stage tumors. While traditional clinicopathological classification systems remain important, recent advances in molecular profiling have improved the understanding of the biological diversity underlying this disease. Genomic alterations, epigenetic dysregulation, neuroendocrine-associated transcriptional programs, and tumor microenvironmental features may contribute to differences in tumor behavior and therapeutic response. Emerging therapeutic approaches, including peptide receptor radionuclide therapy, immunotherapy, anti-angiogenic therapy, and other targeted strategies, are currently under investigation and remain largely exploratory, with most evidence derived from small series, case reports, or extrapolation from other neuroendocrine malignancies. However, current evidence remains limited by the rarity of ONB, the predominance of retrospective studies, and the lack of large prospective or molecularly characterized cohorts. Consequently, many proposed biomarkers and targeted therapeutic strategies remain investigational and require further validation. Future progress in ONB management will depend on integrating molecular characterization with clinical outcome data to improve disease stratification and facilitate the development of biomarker-guided, individualized therapeutic approaches.

## Figures and Tables

**Table 1:** Relationship between the proposed molecular functional states in this review and previously published molecular classification frameworks of ONB.

Proposed Molecular Functional State	[13]	[15]	[16]	Interpretation
Proliferative	Basal-like subgroup characterized by proliferative and progenitor-like features	Developmental programs associated with proliferative olfactory progenitor cells	Basal subtype within the NBM classification	Conceptual functional state integrating proliferative transcriptional programs reported across recent studies.
Neuroendocrine Differentiated	Neural-like subgroup with neuronal differentiation features	Neuroendocrine developmental programs involving OTX2, ASCL1, NEUROD1 and neuronal differentiation pathways	Neural subtype within the NBM classification	Conceptual functional state integrating neuroendocrine differentiation programs reported across recent studies.
Mesenchymal and Stromal-Associated	Not identified	Not specifically described	Mesenchymal subtype characterized by mesenchymal, ECM, angiogenic and stromal-related transcriptional programs	Conceptual functional state proposed in this review based primarily on the mesenchymal subtype described by Yang et al. (2024).

***Footnote:*** *The proposed molecular functional states represent a conceptual integration of transcriptional programs described in previous molecular classification studies and do not represent formally established molecular subtypes. **Abbreviations:** ONB, olfactory neuroblastoma; NBM, Neural–Basal–Mesenchymal; OTX2, Orthodenticle Homeobox 2; ASCL1, Achaete-scute homolog 1; NEUROD1, Neurogenic Differentiation 1.*

**Table 2:** Molecular alterations and biological features reported in ONB.

Category	Representative Findings	Biological Relevance	References
Genomic alterations	IDH2 mutations, chromosomal instability, copy-number alterations, TP53 mutations, Alterations in PI3K/AKT/mTOR pathway, DMD deletions, NOTCH pathway dysregulation	Molecular heterogeneity and altered tumor biology	[12,13,21,22,35]
Epigenetic dysregulation	DNA methylation changes; EZH2-associated transcriptional regulation	Altered transcriptional regulation and tumor progression	[20,36,46,47,48]
Neuroendocrine differentiation	Neuroendocrine markers: INSM1, CHGA, SYP, CD56 Transcription factors: ASCL1, NEUROD1, OTX2	Supports neuroendocrine lineage characteristics of ONB	[14,15,49,50]
Proliferative signaling	Cell-cycle-related transcriptional programs and increased proliferative activity	Associated with aggressive clinicopathologic behavior	[13,14,16,49]
Tumor microenvironment	Variable immune infiltration and stromal composition	Potential influence on immune activity and therapeutic responsiveness	[17,18,19,28,29]
Angiogenesis-associated signaling	Increased microvessel density, VEGF expression, and angiogenesis-related signaling	Potential role in tumor progression and aggressive disease behavior	[28,51]
Stromal remodeling	ECM organization and stromal-associated transcriptional programs	Tumor heterogeneity and invasive phenotype	[14,16]
Molecular heterogeneity	Proliferative, neuroendocrine-differentiated, and stromal-associated transcriptional states	Provides insight into subtype-associated biology and intertumoral heterogeneity	[13,14,15,16,24]

***Abbreviations:*** *ONB, olfactory neuroblastoma; IDH2, isocitrate dehydrogenase 2; TP53, tumor protein p53; PI3K, phosphoinositide 3-kinase; AKT, protein kinase B; mTOR, mechanistic target of rapamycin; DMD, dystrophin gene; EZH2, enhancer of zeste homolog 2; INSM1, insulinoma-associated protein 1; CHGA, chromogranin A; SYP, synaptophysin; ASCL1, Achaete-scute homolog 1; NEUROD1, neuronal differentiation 1; OTX2, orthodenticle homeobox 2; VEGF, vascular endothelial growth factor.*

**Table 3:** Neuroendocrine-associated markers and transcription factors in ONB.

Factor	Category	Biological Role	Clinical Significance in ONB	References
Synaptophysin	Neuroendocrine marker	Neuroendocrine differentiation	Widely used diagnostic marker supporting neuroendocrine differentiation	[50]
Chromogranin A	Neuroendocrine marker	Secretory granule protein	Frequently expressed in ONB and used in routine pathological diagnosis	[50]
CD56 (NCAM)	Neuroendocrine marker	Cell adhesion	Common diagnostic marker supporting neuroectodermal origin	[50]
INSM1	Neuroendocrine marker/transcriptional regulator	Lineage-specific transcription	Highly sensitive diagnostic marker; may reflect lineage-specific transcriptional programs	[50,58,59]
OTX2	Developmental transcription factor	Maintains neuroendocrine lineage	Frequently overexpressed in ONB; may maintain neuroendocrine differentiation	[14,15,22]
ASCL1	Neuroendocrine transcription factor	Neuroendocrine transcription program	Supports neuroendocrine phenotype; biological significance requires further investigation	[14,15,23,49]
NEUROD1	Neuroendocrine transcription factor	Neuronal differentiation	Associated with neuroendocrine differentiation; therapeutic significance remains unclear	[14,15,23,49]

***Abbreviations:*** *INSM1, insulinoma-associated protein 1; OTX2, orthodenticle homeobox 2; ASCL1, Achaete-scute family bHLH transcription factor 1; NEUROD1, neuronal differentiation factor 1; NCAM, neural cell adhesion molecule.*

**Table 4:** Emerging therapeutic strategies and associated biomarkers in recurrent or advanced 601 ONB.

Therapeutic Strategy	Representative Approaches	Biological Rationale	Current Evidence in ONB	References
PRRT	177Lu-DOTATATE; 90Y-DOTATOC	Somatostatin receptor expression, particularly SSTR2	Preliminary clinical activity has been reported in selected patients	[70,71,73,74,75]
Anti-angiogenic therapy	Bevacizumab	VEGF-associated angiogenic signaling	Limited evidence from case reports	[28,29,51]
Epigenetic-targeted approaches	Histone modification and DNA methylation-targeted approaches	IDH2-associated and epigenetic dysregulation	Primarily investigational	[22,36,52,54,55]
Immune checkpoint blockade	Pembrolizumab, nivolumab	PD-L1 expression and immune-cell infiltration	Limited evidence from case reports and retrospective studies	[17,18,30,66]

***Footnote:*** *The therapeutic strategies summarized in this table include approaches supported by ONB-specific evidence as well as strategies extrapolated from related malignancies. Given the rarity of ONB, most targeted therapeutic approaches remain investigational and require further validation in molecularly characterized clinical cohorts. **Abbreviations:** ONB, olfactory neuroblastoma; PRRT, peptide receptor radionuclide therapy; SSTR2, somatostatin receptor 2; PD-L1, programmed death-ligand 1; VEGF, vascular endothelial growth factor; IDH2, isocitrate dehydrogenase 2.*

## Data Availability

Data sharing is not applicable to this article as no datasets were generated or analyzed during the current study. All data supporting the findings of this review are available within this article and its references.
